# Mathematical models used to inform study design or surveillance systems in infectious diseases: a systematic review

**DOI:** 10.1186/s12879-017-2874-y

**Published:** 2017-12-18

**Authors:** Sereina A. Herzog, Stéphanie Blaizot, Niel Hens

**Affiliations:** 1 Institute for Medical Informatics, Statistics and Documentation, Medical University of Graz, Graz, Austria; 2 Centre for Health Economics Research and Modelling Infectious Diseases (CHERMID), Vaccine and Infectious Disease Institute (VAXINFECTIO), University of Antwerp, Antwerp, Belgium; 3 Interuniversity Institute for Biostatistics and statistical Bioinformatics, Hasselt University, Hasselt, Belgium

**Keywords:** Study design, Research design, Mathematical models, Infectious diseases, Systematic review

## Abstract

**Background:**

Mathematical models offer the possibility to investigate the infectious disease dynamics over time and may help in informing design of studies. A systematic review was performed in order to determine to what extent mathematical models have been incorporated into the process of planning studies and hence inform study design for infectious diseases transmitted between humans and/or animals.

**Methods:**

We searched Ovid Medline and two trial registry platforms (Cochrane, WHO) using search terms related to infection, mathematical model, and study design from the earliest dates to October 2016. Eligible publications and registered trials included mathematical models (compartmental, individual-based, or Markov) which were described and used to inform the design of infectious disease studies. We extracted information about the investigated infection, population, model characteristics, and study design.

**Results:**

We identified 28 unique publications but no registered trials. Focusing on compartmental and individual-based models we found 12 observational/surveillance studies and 11 clinical trials. Infections studied were equally animal and human infectious diseases for the observational/surveillance studies, while all but one between humans for clinical trials. The mathematical models were used to inform, amongst other things, the required sample size (*n* = 16), the statistical power (*n* = 9), the frequency at which samples should be taken (*n* = 6), and from whom (n = 6).

**Conclusions:**

Despite the fact that mathematical models have been advocated to be used at the planning stage of studies or surveillance systems, they are used scarcely. With only one exception, the publications described theoretical studies, hence, not being utilised in real studies.

**Electronic supplementary material:**

The online version of this article (10.1186/s12879-017-2874-y) contains supplementary material, which is available to authorized users.

## Background

Infectious diseases contribute substantially to the global burden of disease and are major public health issues worldwide [1]. The value of mathematical models for infectious diseases is widely recognised in various fields such as ecology or epidemiology [2–4]. These models have been used with different objectives such as understanding infectious disease dynamics, informing public health policies or guidelines through, for example, the modelling of potential additional interventions [5], or computing key indicators [6]. In the context of public health surveillance in particular, mathematical models have been also used to detect potential epidemics making use of (temporal) surveillance system data [7], evaluate the performances of a surveillance system [8], or monitor programmes [9].

Mathematical models may also help in informing design of studies, including cross-sectional studies, clinical trials, or surveillance systems, and have been advocated to be used at the planning stage of studies to inform their design [5, 10–13]. Well-designed clinical trials and observational studies are needed to investigate the impact of interventions before they can be used on large scale. Furthermore, public health authorities also need efficient tools for monitoring infectious diseases. Each improvement in design and monitoring of clinical trials and observational studies will allow a more efficient usage of resources crucial to current and later implementation, monitoring, and evaluation of promising interventions.

The current use of mathematical models in planning studies has to our knowledge never been systematically summarised. The objective of our study was to systematically review mathematical models used to inform the design of a study related to an infectious disease transmitted between humans, between animals, or between animals and humans (zoonosis). We documented to which extent and how mathematical models have been incorporated into the process of planning studies or surveillance systems. Finally, by performing this review, we hope to trigger more attention to the use of mathematical models in planning studies and to more explicitly document design considerations in mathematical modelling studies in abstract and keywords.

## Methods

We used a protocol to describe the methods in detail for our systematic review (Additional file 1).

### Eligibility criteria

We searched for publications and registered trials in which mathematical models were described and used to inform the design of infectious disease studies, i.e. inform study design in the context of sample sizes and/or selecting the (number of) sampling times and/or power calculation and/or from whom to collect samples and/or what should be monitored. We were interested in implemented studies as well as in methodological papers.

We included studies that used individual-based models (IBMs, including agent-based models, microsimulation, etc.), compartmental models, or Markov models [14, 15]. IBMs are models in which the infection process for every individual in the population is tracked; compartmental models are models in which individuals in the population are subdivided into ‘compartments’ and the models track the infection process for these individuals collectively; and Markov models predict how an individual moves from one health state to another over time, assuming that the individual is always in one of a finite number of states and that the transition to the next state depends only on the values of the current state.

We excluded publications and study protocols of registered trials if the mathematical model was not used to design a study, for example, the model was only used for data analysis or only used for investigating the potential impact of new interventions on infectious disease spread.

### Information source

We searched Ovid Medline, Cochrane Central Register of Controlled Trials, and WHO International Clinical Trials Registry Platform from the earliest date of the database to October 2016 without language restrictions. Search strategies used subject headings specific to each database and free text search that combined terms for: infection, mathematical models, and study designs (see Appendix 1 in Additional file 1 for the detailed search strategies). Reference lists of included publications and study protocols of registered trials were screened to identify additional relevant publications.

### Selection

Two reviewers (SH, SB) screened titles and abstracts of retrieved publications and description of registered trials in the database. Discrepancies were solved by discussion or by consulting a third reviewer (NH). Any publication or registered trial selected as being potentially eligible was retained for review of the full text; for registered trials we made three attempts to retrieve the study protocol by contacting electronically the principal investigator (listed in the trial registry).

### Outcomes

The primary outcomes were the description of the characteristics of the mathematical models incorporated, and the description of the design part considered in the process of planning studies or surveillance systems.

### Data collection and analysis

The two reviewers (SH, SB) independently extracted data and discrepancies were solved by discussion or by consulting the third reviewer (NH). An extraction sheet was developed using Microsoft Excel and piloted to extract information about the investigated infection, population, model characteristics, and study design.

The characteristics of mathematical models used in planning studies were summarised stratified by study type, i.e. observational and surveillance studies, and clinical trials. We described the infections and populations studied, the main characteristics of the mathematical model used, the main outcome of the study, and the design outcomes (see Table 1) investigated by the authors. If there were multiple publications using the same model for the same setting to investigate the same study design, the earliest publication was considered to be the original (see further). We also documented model reporting items (e.g. diagram of the model or parameters values) and how the infection transmission was modelled.

**Table 1:** Description of design outcomes

Design outcome	Description
Follow-up	The model was used to determine/inform the follow-up time of the study.
Timing of sampling	The model was used to determine/inform at which time point(s) sampling should be performed.
Frequency	The model was used to determine/inform the frequency at which sampling has to be collected (over time) during the study.
Number	The model was used to determine/inform the number of sampling to collect over time during the study.
Monitoring	The model was used to identify parameters or indicators that should be monitored during the study.
Sample size	The model was used to determine/inform the sample size.
Whom	The model was used to determine/inform which subgroups of the population studied should be sampled.
Power	The model was used to perform statistical power calculations.

## Results

**Fig. 1:**
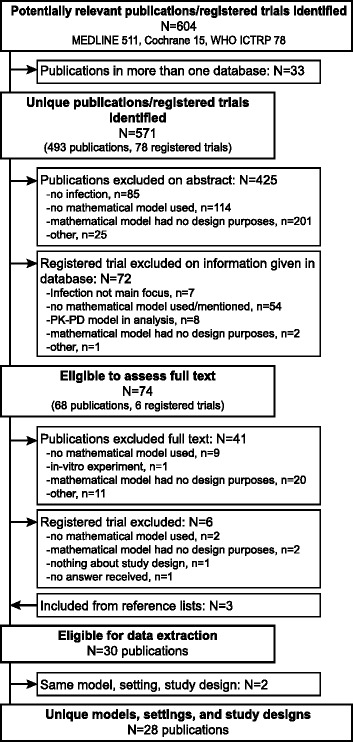
Flow diagram of included and excluded publications and registered trials

Our literature searches identified 571 unique publications/registered trials; 68 full-text publications were screened and 6 principal investigators of registered trials were contacted; 30 eligible publications were included [16–45]. Reasons for exclusion are summarised in Fig. 1. Of these 30 publications, 28 were considered to be unique, i.e. different models were used for different settings and study designs. There were two publications referring to the same randomised controlled trial (RCT) and reporting the same model (Cori 2014 [35] and Hayes 2014 [40]), and one publication re-stated the results from a previous publication (Wu 2005 [39] and Wu 2002 [30]).With only one exception (Cori 2014 [35]), the publications described theoretical studies, hence, not being utilised in real trials.

We focused on the characteristics of the 23 studies which used IBMs or compartmental models to inform the study design because, in the context of infectious diseases, these types of models are most used and allow infectious disease dynamics (e.g. herd immunity) to be directly incorporated. There were 12 observational or surveillance studies [16–27] (Table 2, Additional file 2: Table S1) and 11 clinical trials [28–38] (Table 3, Additional file 2: Table S2). The characteristics of the 5 studies which used Markov models [41–45] (all clinical trials for humans) are given in Additional file 2 (Table S3 and Table S4).

**Table 2:** Characteristics of publications – Observational and surveillance studies (*N* = 12)

First author, year	Infection		Population	Model		Main outcome	Design outcome(s)	Remarks
	Epidemiological category	Name		Type^a^	Structured/Network^b^			
Graat, 2001 [16]	Animal	Bovine herpesvirus 1	Cattle farming	Compartmental - deterministic	Yes/No	Reproduction ratio between herds	- Frequency - Sample size	−
Michael, 2006 [17]	Human, vector-borne	Lymphatic filariasis	Not described	Compartmental – deterministic^c^	No/No	Prevalence of microfilaraemia	- Frequency - Sample size - Monitoring - Power	−
Savill, 2008 [18]	Animal	Avian influenza	Commercial poultry flocks (The Netherlands)	IBM	Yes/No	False alarm rate	- Monitoring	−
Arnold, 2013 [19]	Animal	Avian influenza	Poultry farming	IBM	Yes/Yes	Size and duration of an outbreak	- Sample size - Whom	Spatial model
Smieszek, 2013 [20]	Human, respiratory	Influenza	An US high school (teachers, students, staff)	IBM	Yes/Yes	Performance of collocation ranking	- Sample size - Whom	−
Ciccolini, 2014 [21]	Human, nosocomial	Nosocomial pathogens	Acute hospitals (England, The Netherlands)	Compartmental - stochastic	Yes/Yes	Time to detection and number of infected hospitals	- Sample size - Whom	−
Gonzales, 2014 [22]	Animal	Avian influenza	Layer chickens (The Netherlands)	Compartmental - deterministic	Yes/No	Required sample size and frequency for early detection	- Frequency - Number - Sample size	−
Leslie, 2014 [23]	Animal	Classical swine fever	Wild pig, Kimberley region (Australia)	IBM^c^	Yes/Yes	Epidemic length, number of days to complete the surveillance, number of cells sampled, number of groups to be sampled	- Sample size - Whom	A within-herd model combined with a spatial between-herd model
Mizumoto, 2014 [24]	Human, vector borne	Dengue virus	Not described	Compartmental - deterministic	Yes/No	Relative risk of severe dengue and ‘dengue hemorrhagic fever’/ ‘dengue shock syndrome’ during secondary infection	- Timing of sampling	−
Pinsent, 2014 [25]	Animal	Avian influenza	Commercial poultry barns	Compartmental - deterministic	No/No	Estimates of basic reproduction number and time of virus introduction	- Frequency - Sample size	−
van Bunnik, 2015 [26]	Human, nosocomial	Meticillin-resistant *Staphylococcus aureus*	Hospitals (Scotland)	Compartmental - stochastic	Yes/Yes	Time until first detection of new health-care associated infection	- Sample size - Whom	Similar model as Ciccolini, 2012
Vinh, 2015 [27]	Human, respiratory	Influenza	General population	Compartmental - deterministic	No/No	Statistical identifiability of antibody generation, antibody waning, and reinfection	- Frequency - Sample size - Power	−

^a^model type: IBM – individual based model; ^b^ structured: population structure is reflected in model, network: network of contacts between individuals is explicitly modelled; ^c^ model type obtained from the original article

**Table 3:** Characteristics of publications – Clinical trials (*N* = 11)

First author, year	Infection		Population	Model		Main outcome	Design outcome(s)	Remarks
	Epidemiological category	Name		Type ^a^	Structured/Network ^b^			
Atlas, 1993 [28]	Human, water-borne	*Cryptosporidium*	Volunteer subjects	Compartmental - deterministic^c^	No/No	Probability of infection	- Sample size	Exprimental study
Lipsitch, 2001 [29]	Human, respiratory	*Streptococcus pneumoniae*	Not described	Compartmental - deterministic	No/No	Simple and conditional odds-ratios	- Timing of sampling	−
Wu, 2002 [30]; Wu, 2005 [39]	Human, STI	HIV	Within-host (cells)	Compartmental - deterministic	No/No	HIV viral load change	- Timing of sampling - Frequency - Number - Sample size - Power	Statistical model used for fitting data
Clermont, 2004 [31]	Human, bacterial	Generic Gram-negative pathogen	Within-host (virtual infected patients)	Compartmental - deterministic	Yes/No	Identify people who will well respond to the anti-tumor necrosis factor	- Whom	−
Hallett, 2008 [32]	Human, STI	HIV	Heterosexual population	Compartmental - deterministic	Yes/Yes	HIV incidence rate ratio	- Follow-up - Sample size - Power	−
Dimitrov, 2013 [33]	Human, STI	HIV	Heterosexual population representative of sub-saharan Africa	Compartmental - deterministic	Yes/No	HIV incidence	- Sample size - Power	−
Nishiura, 2013 [34]	Animal	influenza A viruses	Ferret in cages	Compartmental - stochastic	No/No	Number of pairs to include in 1-to-1 transmission studies	- Sample size - Power	−
Cori, 2014 [35]; Hayes, 2014 [40]	Human, STI	HIV	Adults, 18-44y, South Africa and Zambia	Compartmental - deterministic	Yes/Yes	HIV incidence	- Monitoring - Power	Effectively used to plan a three-arm cluster RCT
Cuadros, 2014 [36]	Human, STI	HIV	Serodiscordant couples; male population	IBM	No/Yes	HIV incidence	- Power	−
Scott, 2014 [37]	Human, respiratory	*Streptococcus pneumoniae*	Infants	Compartmental - deterministic	Yes/No	Vaccine efficacy against acquisition and/or duration	- Follow-up - Timing of sampling - Monitoring	−
Herzog, 2015 [38]	Human, STI	*Chlamydia trachomatis*	Women	Compartmental - deterministic	No/No	Pelvic inflammatory disease incidence	- Follow-up - Sample size - Power	−

^a^model type: IBM – individual based model; ^b^ structured: population structure is reflected in model, network: network of contacts between individuals is explicitly modelled; ^c^ model seen as compartmental model

Results of the considered 23 studies show that compartmental models were the most commonly used models (8 observational/surveillance studies, 10 clinical trials). Infections studied were equally animal and human infectious diseases for the observational or surveillance studies, while all but one between humans for clinical trials. Epidemiological categories of infections transmitted between humans were sexually transmitted infections (STIs, mainly HIV; 6 clinical trials), respiratory infections (2 observational/surveillance studies, 2 clinical trials), nosocomial infections (2 observational/surveillance studies), vector-borne infections (2 observational/surveillance studies), water-borne infections (1 observational/surveillance study), hypothetical bacterial infection (1 observational/surveillance study). Influenza (avian, *n* = 4, or in ferrets, *n* = 1) was the infection the most studied among infections transmitted between animals. A population structure was reflected in 14 models (9 observational/surveillance studies, 5 clinical trials) and a network of contacts between individuals was explicitly modelled in 8 models (5 observational/surveillance studies, 3 clinical trials).

We observed diverse patterns of model reporting across the publications (Additional file 2: Table S1 and Table S2). Almost all publications described - to a certain extent - the model structure, some reported equations, figures, and how the course of infection was implemented, while others reported only one or even none of those. For three publications [17, 23, 28] we had to obtain the model type from the referenced original publication [46–48]. About a third of the publications reported the software in which the mathematical model was implemented but code is available, either as a supplementary material or through request to the authors, in only two publications [36, 38]. The sources for the model parameters used were mostly a mixture of calibration, assumptions by authors, and estimations from other data.

In observational or surveillance studies, the design outcome most studied was sample size (*n* = 10), followed by frequency of sampling and population from whom to sample (*n* = 5), monitoring and power (*n* = 2), and number of samples and timing of sampling (n = 2). In clinical trials, the most studied design outcome was power (*n* = 7), followed by sample size (*n* = 6), timing of sampling and follow-up (*n* = 3), monitoring (n = 2), frequency of sampling, number of samples and population from whom to sample (n = 1). Seven research question categories were identified among the studies included: detect infection early, estimate epidemiological parameters, compare different trial arms, include potentially good responders in an RCT, follow trial progression, detect changes in infection values over time, and determine appropriate time point(s) to estimate a parameter. Table 4 shows for each design outcome which research questions were investigated. More details about the study designs can be found in Additional file 2: Table S1 and Table S2.

**Table 4:** Design outcomes and corresponding research question

Design outcomes	References by main research questions
Follow-up	Determine appropriate time point to estimate a parameter: Mizumoto [24]; Lipsitch [29]; Wu [30]; Scott [37]; Herzog [38]; Hallett [32]
Timing of sampling	Determine appropriate time point to estimate a parameter: Mizumoto [24]; Lipsitch [29]; Wu [30]; Scott [37]; Herzog [38]; Hallett [32]
Frequency	Detect infection early: Graat [16]; Michael [17]; Gonzales [22] Estimate epidemiological parameters: Pinsent [25]; Vinh [27] Compare different trial arms: Wu [30]
Number	Detect infection early: Gonzales [22] Compare different trial arms: Wu [30]
Monitoring	Detect infection early: Michael [17]; Savill [18] Follow trial progression: Cori [35]; Scott [37]
Sample size	Detect infection early: Graat [16]; Michael [17]; Arnold [19]; Smieszek [20]; Ciccolini [21]; Gonzales [22]; Leslie [23]; van Bunnik [26] Estimate epidemiological parameters: Atlas [28]; Pinsent [25]; Vinh [27] Compare different trial arms: Wu [30]; Hallett [32]; Dimitrov [33], Nishiura [34]; Herzog [38]
Whom	Detect infection early: Arnold [19]; Smieszek [20]; Ciccolini [21]; Leslie [23]; van Bunnik [26] Include potentially good responders in a RCT: Clermont [31]
Power	Estimate epidemiological parameters: Vinh [27] Compare different trial arms: Wu [30]; Hallett [32]; Dimitrov [33]; Nishiura [34]; Cori [35]; Cuadros [36]; Herzog [38] Detect changes in infection values over time: Michael [17]

## Discussion

We found in this systematic review 28 unique publications but no registered trials in which mathematical models were described and used to inform the design of infectious disease studies. Only one mathematical model was effectively used to plan a study (a three-arm cluster RCT) whereas all others described theoretical studies. Focusing on the 23 compartmental and individual-based models, we found almost equal amount of observational or surveillance studies and clinical trials whereby compartmental models were most commonly used. Various infection categories have been investigated with equal numbers of animal and human infections studied among the observational or surveillance studies and mainly human infections among the clinical trials. Enough details are provided for the compartmental models, except for two [17, 22], to replicate the model. For IBMs more details are needed in order to replicate the model if the source code is not available. For example, the ‘ODD’ (Overview, Design concepts, and Details) protocol has been proposed to standardize reporting of individual-based and agent-based models [49, 50]. None of the five IBM publications followed or mentioned this protocol, however, one provides the model source code [36]. The mathematical models were utilised to inform, amongst other things, the following design outcomes: required sample size, statistical power, frequency at which samples should be taken, and from whom.

One explanation of the scarcity of mathematical modelling to design real studies, despite the anticipated gain in study efficiency, is arguably the lack of fundamental research in the sense that there are no existing databases or software to access mathematical models which are already implemented in a study design framework in contrast to classical sample size calculation with freely-available or chargeable software. In our systematic review, only a third of the publications reported the software used for the mathematical model and only two mentioned availability of code. Additionally, it is even difficult to ascertain which mathematical models already exist for a specific infection in order to extend them to the study design framework. Account should be taken of the fact that building a (well-validated) model is time-consuming and modelling expertise specific to the infectious disease of interest is needed. On the other hand, finding the optimal sample size, frequency of sampling, etc. using a mathematical model can lead to computer intense processing steps.

Sample size is not the only design outcome which can be investigated by the mathematical models as shown in our results. Interestingly, mathematical models have been used to determine the appropriate time point(s) at which a parameter such as the effect size of the intervention should be estimated. For example, a publication in the field of vaccination investigated two different estimates of vaccine efficacy (prevalence odds ratio and prevalence ratios) and their change over time. The authors found that the timing of sample collection can affect the interpretation of results about vaccine efficacy against bacterial carriage in an RCT [37].

We observed that the same research question was investigated looking at different design outcomes. For example, the question about early detection of an infection was studied by exploring the frequency at which samples should be taken, the number of samples to be collected, and the sample size but also from whom samples should be taken, and what parameters or indicators should be monitored during a study [16–23, 26].

There is no guarantee that a study would not fail to show the expected effect size in case the study team used a mathematical model at the planning stage of a study. A mathematical model always implies underlying assumptions by its structure and parameter values. However, those assumptions for mathematical models can be taken into account in uncertainty and sensitivity analyses for the effect size of interest. The estimated effect size and its uncertainty, in turn, can be considered in, for example, sample size calculations. Most importantly, mathematical models can be used to deal with the complexity of infectious diseases like the dependency between individuals and give insights how this can influence the study design.

The strength of our study is that the search of publications and registered trials had no restriction about the type of infection or the transmission route. Additionally, this is, to our knowledge, the first systematic review of the current use of mathematical models in planning infectious disease studies. A weakness of this review is that the literature search might have missed some relevant publications and registered trials because there is neither a single MeSH term for mathematical models nor a general usage of keywords to describe mathematical models, a problem observed also in other systematic reviews [51, 52]. In addition, for some infections, like HIV, only the abbreviation is commonly stated. Hence, no variation of the word ‘infection’ is used in the title, abstract, or especially in the description of the registered trial on the trial registry platform. We tested the inclusion of specific infections (HIV, malaria, and tuberculosis) which resulted in a limited number of additional hits, of which only two would meet our inclusion criteria [53, 54]. We tried to overcome the limitations of keywords and MeSH terms used by building a search strategy which uses different terms to identify models and infections and by searching for additional publications in the reference lists of included publications. However, we do not present mathematical models that are only described in grey literature like reports or that are not publicly available. Similarly, publications that only used indirect modelling results without describing the mathematical model were not included.

## Conclusion

Despite the fact that mathematical models have been advocated to be used at the planning stage of studies or surveillance systems [5, 10–13], they are used scarcely as shown by this systematic review. With only one exception, the publications described theoretical studies, hence, not being utilised in real studies. Generic statistical approaches for sample size calculations, most often assuming independence between individuals, do not capture the complex nature of infectious disease epidemiology. This is an oversimplification as e.g. treating infected persons may affect the people around them by reducing the spreading of the infection; mathematical modelling could thus be used to account for such characteristics. The results of this systematic review offer an overview of the current use of mathematical models in the context of study design and indicate that future research is needed.

## Additional files

Additional file 1: Study protocol and search strategy. (PDF 74 kb)

Additional file 2: Additional results tables. (PDF 54 kb)

## Abbreviations

IBM: Individual-based model
RCT: Randomised controlled trial
STI: Sexually transmitted infection
