# Supplementary material for: Mathematical models used to inform study design or surveillance systems in infectious diseases: a systematic review
Source: BMC Infect Dis. 2017 Dec 18;17:775. doi: 10.1186/s12879-017-2874-y (PMC5735541; doi:10.1186/s12879-017-2874-y)
Supplement: Supplementary file 1 — Study protocol and search strategy. (PDF 74 kb) [file 12879_2017_2874_MOESM1_ESM.pdf]

Mathematical models used to inform study design or surveillance systems in infectious diseases: a systematic review

Sereina A. Herzog, Stéphanie Blaizot, Niel Hens

Additional File 1
Protocol used for the systemic review

Protocol for the systematic review of mathematical models used to inform study design or surveillance systems in infectious diseases

Contents

Contents 2
1. Background 2
2. Objective 3
3. Methods 3
3.1. Questions 3
3.2. Inclusion criteria 3
3.3. Exclusion criteria 4
3.4. Search strategy 4
3.5. Selection and retrieval of publications 5
3.6. Data extraction forms 6
3.7. Data extraction 6
3.8. Data analysis 6
3.9. Assessment of quality of reporting 6
4. Write report 6
5. References 7
6. Appendix 1: search strategies 8
6.1. Medline 8
6.2. Cochrane 9
6.3. WHO International Clinical Trials Registry Platform 9

1. Background

Infectious diseases contribute substantially to the global burden of disease [WHO 2013]. Well-designed randomized controlled trials (RCTs) and observational studies are needed to investigate the impact of interventions before they can be used on large scale. Furthermore, public health authorities also need efficient tools to monitor infectious diseases. Each improvement in design and monitoring of clinical trials and observational studies will allow a more efficient usage of resources crucial to current and later implementation, monitoring, and evaluation of promising interventions. One important part at the planning stage of a study is the estimation of the required sample size as well as selecting the (number of) sampling times. Indeed, sample size and sampling times raise special issues in the field of infectious diseases: the intervention (e.g., new treatment) affects not only the individuals in a study but also people around them. For example, a successfully treated person infects fewer people due to, say, shortened infection duration and therefore the treatment also indirectly protects susceptible persons. Mathematical models offer the possibility to investigate the dynamic response of the infectious disease pressure which results from an intervention.

Mathematical models have been applied to many infectious diseases in humans but also in disease dynamics in animals and zoonoses. Mathematical models are used to improve our understanding of infectious disease epidemiology using e.g. serological or incidence data with or without social contact data in order to estimate epidemiological parameters [Hens 2012]. They are also used to complement statistical approaches for the data analysis such as in cluster RCTs in HIV [Boily 2012]. Mathematical models have been advocated to be used at the planning stage of studies to inform the study design of preventive interventions for infectious diseases [Boily 1999, Boily 2008, Boily 2012, Garnett 2011, Wang 2014, Herzog 2015].

The current use of mathematical models in planning studies has to our knowledge never been systematically summarized. We will be able to show to what extent mathematical models have been incorporated into the process of planning infectious disease studies by searching for methodological papers, implemented studies as well as for study protocols (from trial registries).

2. Objective

To conduct a systematic review in order to determine to what extent mathematical models have been incorporated into the process of planning studies or surveillance systems and hence inform study design for infectious diseases transmitted between humans, between animals, and between animal and human (zoonosis).

3. Methods

This section describes the research questions and the methods for preparing the systematic review.

3.1. Questions

Questions to be answered by the systematic review

- How are mathematical models used to inform study design in the process of planning infectious disease trials or surveillance systems?
- Which outputs of a mathematical model are used to inform sample size consideration and/or (number of) sampling times?

3.2. Inclusion criteria

We will search for publications and registered trials in which mathematical models are used to inform the study design of infectious disease studies, i.e. clinical trials, observational studies, or surveillance systems. The mathematical model or output from the mathematical model should be used to calculate e.g. required sample sizes and/or selecting the (number of) sampling times. We are interested in implemented studies as well as in methodological papers.

3.2.1. Infection

We will consider the following infections for inclusion

- Infectious diseases transmitted between humans, between animals, and between animal and human (zoonosis)

3.2.2. Model types

We will consider following mathematical models for inclusion. As described by Vynnycky and White [Vynnycky 2010]

- *Compartmental models* in which individuals in the population are subdivided into ‘compartments’ and the model tracks the infection process for these individuals collectively.
- *Individual-based* (including agent-based, …) or *microsimulation model* in which the model tracks the infection process for every individual in the population.

and also

- *Markov models* predict how an individual moves from one health state to another over time, assuming that the individual is always in one of a finite number of states and that the transition to the next state depends only on the values of the current state [Sonnenberg 1993].

3.2.3. Study types

We will consider the following study types for inclusion

- Observational studies
- Randomized controlled trials
- Surveillance systems

3.2.4. Outcomes reported

One or more of the following information has to be reported as an output/result of the mathematical model to inform the study design (e.g. by using in sample size calculations the effect size estimated from the mathematical model)

- Required sample size
- Number of sampling
- Timing of sampling
- Power calculation

For the study type surveillance system, a publication will be considered eligible if the mathematical

model was used to determine an optimal surveillance strategy (in terms of time to detect infection/outbreaks/first cases, number of units infected, R_0, outbreak duration) or test a surveillance system. More precisely, a paper will be considered eligible if at least one of the following objectives is studied:

- To assess the number of sampling (when)/sampling frequencies and number of individuals in hospitals/livestock holdings/etc. required over a time period to detect cases, an outbreak, …
- To determine the sample size / statistical power for hypothesis testing or identifiability of one or more parameters
- To determine which units (e.g. individuals, hospitals, holdings) have to be surveyed and their sample size
- To assess a given (current) surveillance strategy and compare it with alternative strategies

3.3. Exclusion criteria

We will exclude publications if one or more of the following is applicable:

- The publication is a conference proceeding or a review paper – except the review paper investigates the use of mathematical models in planning studies
- Registered trials if no contact information is given in the registry database and no publication can be found using the trial registry identification number
- In-vitro experiments
- Publications which do not explicitly investigate how a new or better intervention or screening scheme can be validated by a study using a mathematical model to inform the study design. For example, we are not interested in
    - publications having statements like "this mathematical model can be used to inform policy, future interventions,…" but not investigating how a mathematical model can be used to inform the study design;
    - mathematical models investigating when/where in the course of an infection a drug/intervention should aim at but not looking at study design issues;
    - mathematical models investigating which treatment regime (timing) is best except they look also at how to design a study using a mathematical model.
- Publications in the context of surveillance systems in which a model was used to assess only the impact of an intervention/programme (e.g. impact of vaccination) on the spread of an infection cannot be considered eligible, unless different surveillance strategies are compared as well
- Publications/registered trials which will use a mathematical model only for analysis

3.4. Search strategy

The following databases will be searched from the earliest date of the databank to 06.10.2016 without language restrictions (see Appendix 1 for details)

- Ovid Medline
- Cochrane Central Register of Controlled Trials (www.onlinelibrary.wiley.com/cochranelibrary/search)
- WHO International Clinical Trials Registry Platform (ICTRP) which currently (October 2016) includes the following 17 trial registries (http://www.who.int/ictrp; http://apps.who.int/trialsearch/)
    - Australian New Zealand Clinical Trials Registry
    - Chinese Clinical Trial Registry
    - ClinicalTrials.gov
    - EU Clinical Trials Register (EU-CTR)
    - ISRCTN
    - The Netherlands National Trial Register
    - Brazilian Clinical Trials Registry (ReBec)
    - Clinical Trials Registry - India
    - Clinical Research Information Service - Republic of Korea
    - Cuban Public Registry of Clinical Trials
    - German Clinical Trials Register
    - Iranian Registry of Clinical Trials
    - Japan Primary Registries Network
    - Pan African Clinical Trial Registry
    - Sri Lanka Clinical Trials Registry
    - Thai Clinical Trials Register (TCTR)
    - Peruvian Clinical Trials Registry (REPEC)

3.4.1. Search terms

There is no single Medical Subject Heading search term in Medline for mathematical models. Search strategies will use subject headings specific to each database and free text search that combine terms for (see Appendix 1 for details)

- Infection
- Mathematical models
- Study design (including sample size and sampling time)

3.4.2. Additional searches

The following additional searches will be done:

- Reference lists of included publications will be screened

3.5. Selection and retrieval of publications

We use the term ‘publication’ in this section combining ‘publication’ and ‘study protocols’ of registered trials.

In the context of registered trials, the study protocol of the registered trial will be retrieved for review. The study protocol is either available directly by a link on the trial registry homepage, otherwise we make three attempts to contact the principal investigator (listed in the trial registry) by mail. In addition, Ovid Medline will be searched for publication about the registered trial by using the trial registry identification number.

3.5.1. Selection of eligible publications

Two suitably qualified reviewers will review the lists of publications identified by the search strategy independently using the inclusion and exclusion criteria listed in the paragraphs above. Any publication selected as being potentially eligible by either reviewer, will be retained for review of the full text.

3.5.2. Potentially eligible publications

The reviewers will read the abstract of each identified publication if fewer than 600 publications are returned in total. If the searches identify 600 or more publications, the reviewers will select potentially eligible titles first and will then read the abstracts of titles that potentially fit the inclusion criteria. If no abstract is available electronically, the full text of the publication will be requested. The abstracts of publications identified through additional searches (paragraph Additional searches) will be reviewed in the same manner as for studies identified through database searches.

3.5.3. Retrieval of full-text articles

We will obtain the full text of publications or other documents reporting publications identified as being potentially eligible for inclusion. We will make every effort to locate documents through internet downloads, inter-library loans and contacting authors of reviews citing potentially eligible documents. We will request translation if necessary to confirm or refute eligibility.

3.5.4. Selection of publications for inclusion

The two independent reviewers will examine full text publications and compare their lists of publications eligible for inclusion. Publications identified by both reviewers as being eligible for inclusion and having adequate data for extraction will be included in the review. Where there are discrepancies, the reasons for these will be discussed and a decision about inclusion reached by consensus. If there is no agreement, a third independent reviewer will adjudicate to make a final decision about eligibility.

3.6. Data extraction forms

We will develop forms for extracting consistent data about:

- Infection and population investigated with the model
- Description of the mathematical model, i.e. model characteristics
- What information of mathematical model is/are used to inform study design and how this is done
- Information if mathematical model was used in the end to implement a study or if it is methodological work
    - In the case of implemented studies, information about study results including information of type of data (e.g. serological data)

We will develop an extraction form using Excel, Epidata, or another suitable program. We will pilot test the forms to ensure ease of use and capture of all relevant data.

3.7. Data extraction

Two appropriately qualified people will extract and enter data independently from each included study. Articles in languages other than English will either be translated first and then duplicate data extraction conducted as above or, if there are two reviewers who understand the language of the publication, they will extract the data directly.

Discrepancies in data extraction or data entry will be resolved by consensus. If there is no agreement a third independent reviewer will adjudicate to make a final decision.

Studies might be excluded at the data entry stage if it becomes apparent that inclusion criteria are not met or there is not enough information in the documents to extract the required data.

3.8. Data analysis

The data analysis will be descriptive including:

- A flow chart describing included and excluded publications/registered trials
- A table with the characteristics of the included publications/registered trials with information
    - about the model characteristics
    - about the described setting of the study
    - about how model was used to inform study design

3.9. Assessment of quality of reporting

We want to assess the quality of how the mathematical model, which is used to inform the study design or surveillance system, is reported. We will search for checklists of items associated with methodological and reporting quality that are specific to modelling papers – if not, define our own:

- Are the mathematical equations stated which describe the model?
- Is a flow diagram of model shown?
- Is it stated in which languages/programme the model is implemented?
- Is the code available?
- Is an overview given of the parameters used?

4. Write report

A descriptive data analysis will be done after the systematic search has been conducted according to the protocol. The report will be written following the PRISMA Guidelines for reporting of systematic reviews and meta-analyses [Liberati 2009, Moher 2009] and will clearly present the methods used as well as findings.

5. References

- ***Boily 1999:**. Boily MC, Mâsse BR, Desai K, Alary M, Anderson RM. Some important issues in the planning of phase III HIV vaccine efficacy trials. Vaccine. 1999; 17:989-1004.*
- ***Boily 2008:** 26. Boily M, Abu-Raddad L, Desai K, Masse B, Self S, Anderson R. Measuring the public-health impact of candidate HIV vaccines as part of the licensing process. Lancet Infect Dis. 2008; 8:200-207.*
- ***Boily 2012**: Boily M, Mâsse B, Alsallaq R, Padian NS, Eaton JW, Vesga JF, Hallett TB. HIV treatment as prevention: considerations in the design, conduct, and analysis of cluster randomized controlled trials of combination HIV prevention. PLoS Med. 2012; 9:e1001250.*
- ***Garnett 2011:** Garnett GP, Cousens S, Hallett TB, Steketee R, Walker N. Mathematical models in the evaluation of health programmes. Lancet. 2011; 378:515-525.*
- ***Hens 2012:** Hens N, Shkedy Z, Aerts M, Faes C, Van Damme P, Beutels P. Modeling infectious disease parameters based on serological and social contact data: A modern statistical perspective. New York, NY: Springer; 2012.*
- ***Herzog 2015:** Herzog SA, Low N, Berghold A. Sample size considerations using mathematical models: an example with Chlamydia trachomatis infection and its sequelae pelvic inflammatory disease. BMC Infect Dis. 2015, 15:233*
- ***Liberati 2009:** , Liberati A, Altman DG, Tetzlaff J, Mulrow C, Gøtzsche PC, Ioannidis JP, Clarke M, Devereaux PJ, Kleijnen J, Moher D. The PRISMA statement for reporting systematic reviews and meta-analyses of studies that evaluate healthcare interventions: explanation and elaboration. BMJ. 2009; 339:b2700.*
- ***Moher 2009:** Moher D, Liberati A, Tetzlaff J, Altman DG; PRISMA Group. Preferred reporting items for systematic reviews and meta-analyses: the PRISMA statement. PLoS Med. 2009; 6:e1000097.*
- ***Sonnenberg 1993:** Sonnenberg FA, Beck JR. Markov models in medical decision making: A practical guide. Med Decis Making 1993; 13:322–338*
- ***Vynnycky 2010:** Vynnycky E, White RG. An introduction to infectious disease modelling.New York: Oxford University Press; 2010.*
- ***WHO 2013:** Research for universal health coverage. Geneva: WHO; 2013*

6. Appendix 1: search strategies

This section describes the search strategy for each database separately.

6.1. Medline

Infection

#	Searches	Results
**1.**	infectio$.mp	1609641
**2.**	communicable.mp	52044
**3.**	seroepidemiolog$.mp	19202
**4.**	transmit$.mp	131371
**5.**	transmission$.mp	313973
**6.**	1 or 2 or 3 or 4 or 5	1924310

Mathematical model

#	Searches	Results
**7.**	Models, Theoretical/	125778
**8.**	mathematical adj3 model$.mp	37054
**9.**	Markov Chains/	11636
**10.**	micro simulation$.mp	94
**11.**	compartmental adj3 model$.mp	3199
**12.**	7 or 8 or 9 or 10 or 11	167299

Study design

#	Searches	Results
**13.**	sample size$.mp	48044
**14.**	exp Sample Size/	10820
**15.**	sampl$ tim$.mp	4808
**16.**	sampling.mp	157829
**17.**	sampl$ frequenc$.mp	1176
**18.**	((design or designs) adj10 (surveillance or program or programme or programs or programmes or system or systems or trial or trials or study or studies)).mp	396307
**19.**	exp Epidemiological Methods/	5122184
**20.**	(13 or 14 or 15 or 16 or 17 or 18) and 19	384384

Combination

#	Searches	Results
**21.**	6 and 12 and 20	511

Database: Ovid MEDLINE(R) 1946 to September Week 4 2016
Homepage: http://ovidsp.tx.ovid.com/
Date: 06.10.2016

6.2. Cochrane

Infection

#	Searches	Results
**1.**	infectio*	79117
**2.**	Communicable	737
**3.**	seroepidemiolog*	129
**4.**	transmit*	4328
**5.**	transmission*	7193
**6.**	1 or 2 or 3 or 4 or 5	911934

Mathematical model

#	Searches	Results
**7.**	MeSH descriptor: [Models, Theoretical] this term only	928
**8.**	mathematical near/3 model*	745
**9.**	MeSH descriptor: [Markov Chains] this term only	2153
**10.**	micro simulation*	135
**11.**	compartmental near/3 model*	239
**12.**	7 or 8 or 9 or 10 or 11	4002

Study design

#	Searches	Results
**13.**	sample size*	26610
**14.**	MeSH descriptor: [Sample Size] explode all trees	1235
**15.**	sampl* tim*	40716
**16.**	sampling	13392
**17.**	sampl* frequenc*	9080
**18.**	((design or designs) near/10 (surveillance or program or programme or programs or programmes or system or systems or trial or trials or study or studies))	105139
**19.**	MeSH descriptor: [Epidemiologic Methods] explode all trees	376343
**20.**	(13 or 14 or 15 or 16 or 17 or 18) and 19	71722

Combination

#	Searches	Results
**21.**	6 and 12 and 20	167
**22.**	21 limited to ‘Trials”	15

Database: Cochrane Central Register of Controlled Trials
Homepage: http://onlinelibrary.wiley.com/cochranelibrary/search
Date: 12.09.2016

6.3. WHO International Clinical Trials Registry Platform

Basic search

#	Searches	Results
1.	model* AND infection	78

Database: WHO International Clinical Trials Registry Platform
Homepage: http://apps.who.int/trialsearch/ictrpmob.aspx
Date: 06.10.2016
