# Supplementary material for: Mathematical models used to inform study design or surveillance systems in infectious diseases: a systematic review
Source: BMC Infect Dis. 2017 Dec 18;17:775. doi: 10.1186/s12879-017-2874-y (PMC5735541; doi:10.1186/s12879-017-2874-y)
Supplement: Supplementary file 2 — Additional results tables. (PDF 54 kb) [file 12879_2017_2874_MOESM2_ESM.pdf]

Mathematical models used to inform study design or surveillance systems in infectious diseases: a systematic review

Sereina A. Herzog, Stéphanie Blaizot, Niel Hens

Additional File 2
Additional tables

Contents

eTable

S1	Supplementary characteristics of publications – Observational and surveillance studies (N=12)	2
S2	Supplementary characteristics of publications – Clinical trials (N=11)	4
S3	Characteristics of publications – Clinical trials with Markov model (N=5)	5
S4	Supplementary characteristics of publications – Clinical trials with Markov model (N=5)	6

References 7

eTable S1. Supplementary characteristics of publications – Observational and surveillance studies (N=12)

**First author, year**	**Model reporting**					**Infection in model**		**Study design – details**	**Remarks** [b]
	Equations/ Figure/ Program	Parameters listed/ source [a]	Population characteristics at baseline	Model previously published/ specifically built	Sensitivity analyses	Course of infection	Static or Dynamic		
**Graat, 2001**	Yes/No/No	Yes/Mixture	Yes	No/Yes	Yes	SI	Dynamic	Surveillance system	–
**Michael, 2006**	No/No/No	No/–	–	Yes/No	Yes	–	–	Large-scale community-based intervention programmes; monitoring strategies compared	Model not fully described in the article
**Savill, 2008**	Yes/No/No	Yes/Mixture	No	Yes/No	Yes	Latent, asymptomatic, and symptomatic	Dynamic	Surveillance system	–
**Arnold, 2013**	NA/No/No	Yes/Mixture	Yes	No/Yes	Yes	Within premises: SEIR Between premises: spatial transmission	Dynamic	Nine different surveillance strategies compared	–
**Smieszek, 2013**	NA/No/Yes	Partly/ Mixture	Yes	Yes/No	Yes	SEIR	Dynamic	Sentinel surveillance system	–
**Ciccolini, 2014**	Yes/No/No	Partly/ Mixture	Yes	No/Yes	Yes	SI	Dynamic	Sentinel surveillance system	–
**Gonzales, 2014**	No/No/No	Yes/ Estimated from other data	Yes	Yes/Yes	No	SIR	Dynamic	Surveillance system	–
**Leslie, 2014**	NA/No/Yes	Yes/ Estimated from other data	Yes	Yes/No	No	Susceptible, latent, infectious, immune, dead	Dynamic	Different surveillance strategies compared	–
**Mizumoto, 2014**	Yes/Yes/Yes	Yes/Mixture	Yes	No/Yes	Yes	SIR with W: protected state due to a short-lasting cross-protective immunity.	Dynamic	Prospective cohort study	Equations combined the states of the 2 serotypes
**Pinsent, 2014**	Yes/Yes/Yes	Yes/Mixture	Yes	No/Yes	Yes	SEIR with C (seroconversion state); E,I,C classes divided into 5, 5, 10 compartments	Dynamic	Surveillance system	–
**van Bunnik, 2015**	Yes/No/No	Yes/Mixture	Yes	Yes/Yes	Yes	SI	Dynamic	Sentinel surveillance system (comparison current system vs. 2 other schemes)	Model validated
**Vinh, 2015**	Yes/Yes/Yes	Yes/Mixture	No	Yes/Yes	Yes	Exposed (E), infected (I), recovered individuals who have not yet mounted a full antibody	Dynamic	Serial sero-epidemiological study design	–

First author, year	Model reporting					Infection in model		Study design – details	Remarks [b]
	Equations/ Figure/ Program	Parameters listed/ source [a]	Population characteristics at baseline	Model previously published/ specifically built	Sensitivity analyses	Course of infection	Static or Dynamic		
						response (P), recovered individuals whose specific antibody levels would give a result i in an immunological assay (Ri) [used 10 R compartments]			

NA: not applicable; S: susceptible individuals; E: exposed individuals; I: infected individuals; R: recovered individuals.
[a] parameter source: mixture – mixture of calibration, assumed by authors, and estimated from other data
[b] explicitly mentioned in remarks: if model validation was done; if source code for model is available

eTable S2. Supplementary characteristics of publications – Clinical trials (N=11)

First author, year	Model reporting					Infection in model		Study design – details	Remarks [b]
	Equations/ Figure/ Program	Parameters listed/ source [a]	Population characteristics at baseline	Model previously published/ specifically built	Sensitivity analyses	Course of infection	Static or Dynamic		
**Atlas, 1993**	Yes/No/No	Yes/Assumed by authors	Yes	Yes/Unclear	Yes	Not modelled	–	Experimental study (supplementary analysis for a research protocol)	–
**Lipsitch, 2001**	Yes/Yes/No	Yes/Assumed by author	Yes	Yes/Yes	No	Not colonized, colonized with penicillin-susceptible *S. pneumoniae*, colonized with penicillin-resistant *S. pneumoniae*	Static	Authors suggest this approach can be used for other study types	–
**Wu, 2002,Wu, 2005**	Yes/No/No	Yes/Mixture	No	Yes/Yes	Yes	Productively infected cells; long-lived/latently infected cells	Dynamic	HIV dynamics study	–
**Clermont, 2004**	Yes/No/Yes	Yes/Mixture	Yes	No/Yes	No	Not modelled	–	Modelling of components of the human inflammatory response (within-host)	–
**Hallett, 2008**	Yes/No/No	Yes/Mixture	Yes	Yes/Yes	Yes	HIV negative, HIV acute infection, latent infection, AIDS	Dynamic	Cluster RCT	–
**Dimitrov, 2013**	Yes/Yes/No	Yes/Mixture	Yes	Yes/Yes	Yes	Susceptible, infected with wild-type or drug-resistant HIV, AIDS	Dynamic	Individual RCTs (two interventions modelled separately).	–
**Nishiura, 2013**	Yes/No/No	No/Calibrated	No	Yes/No	No	SI	Dynamic	One-to-one animal transmission experiments	–
**Cori, 2014 Hayes, 2014**	Yes/Yes/No	Yes/Mixture	Yes	No/Yes	Yes	SI extended (with circumcision, antiretroviral therapy, …)	Dynamic	Three-arm cluster RCT	–
**Cuadros, 2014**	NA/Yes/Yes	Yes/Mixture	Yes	Yes/Yes	Yes	SI	Dynamic	Two completed trials discussed: Treatment with (val)acyclovir; Male circumcision	Model source code given in supplemental material
**Scott, 2014**	Yes/Yes/No	Yes/Mixture	Yes	No/Yes	Yes	SIS	Dynamic	Cross-sectional observation in the RCT	Model validated
**Herzog, 2015**	Yes/Yes/Yes	Yes/Mixture	Yes	Yes/Yes	Yes	S-I_1-I_2-S	Static	Re-analysing a RCT	Model source code available by request

NA: not applicable; S: susceptible individuals; E: exposed individuals; I: infected individuals; R: recovered individuals; RCT: randomized controlled (clinical) trials
[a] parameter source: mixture – mixture of calibration, assumed by authors, and estimated from other data
[b] explicitly mentioned in remarks: if model validation was done; if source code for model is available

eTable S3. Characteristics of publications – Clinical trials with Markov model (N=5)

First author, year	Infection		Population	Model		Main outcome	Design outcome(s)	Remarks
	Epidemiological category	Name		Type [a]	Structured / Network [b]			
**Chen, 1999**	Human, respiratory	Epstein-Barr virus	Hypothetical Hong Kong males 40-69y	Markov - deterministic	No/No	Death or surrogate endpoint	- Frequency<br>- Sample size<br>- Power	–
**Longini, 1999**	Human, STI	HIV	Uninfected primary participants and their uninfected steady sexual partnership	Markov - stochastic	Yes/No	Vaccine efficacy: susceptibility and infectiousness	- Frequency	–
**Hoad, 2009**	Human, respiratory	*Mycobacterium tuberculosis*	Adult, data from Asembo and Gem (Western Kenya)	Markov - stochastic	Yes/No	Tuberculosis prevalence	- Sample size	Spatial model
**Tuite, 2011**	Human, STI	Hypothetical STI	Serodiscordant couples	Markov - stochastic	No/No	STI incidence	- Sample size<br>- Power	–
**Auranen, 2014**	Human, respiratory	*Streptococcus pneumoniae*	Infants, children	Markov - stochastic[c]	No/No	Vaccine efficacy	- Timing of sampling<br>- Sample size<br>- Power	–

[a] model type: IBM – individual based model; [b] structured: a population structure is reflected in model, network: a network of contacts between individuals is explicitly modelled; [c] model type obtained from the original article

eTable S4. Supplementary characteristics of publications – Clinical trials with Markov model (N=5)

**First author, year**	**Model reporting**					**Infection in model**		**Study design – details**	**Remarks** [b]
	Equations/ Figure/ Program	Parameters listed/ source [a]	Population characteristics at baseline	Model previously published/ specifically built	Sensitivity analyses	Course of infection	Static or Dynamic		
**Chen, 1999**	Yes/No/No	Yes/ Calibrated	Yes	No/No	Yes	0- No disease; 1- Epstein-Barr Virus infection; 2- Pre-clinical screen-detectable phase; 3- Clinical phase	Static	Hypothetical RCT with a screening vs. no screening regime. Four screening regimes compared.	Infection not the main purpose of the article
**Longini, 1999**	Yes/Yes/No	Yes/ Assumed by author	Yes	No/Yes	Yes	SI	Static	Augmented RCT	
**Hoad, 2009**	Yes/No/No	Yes/ Mixture	Yes	No/No	Yes	SIS	Dynamic	Group or community randomised trial	
**Tuite, 2011**	No/Yes/Yes	Yes/ Mixture	Yes	No/No	No	SI	Static	(Sero-)discordant couple studies	–
**Auranen, 2014**	No/No/No	No/–	No	Yes/Yes	No	–	–	Cross-sectional observation in vaccine trials	Model not fully described in the article

NA: not applicable; S: susceptible individuals; E: exposed individuals; I: infected individuals; R: recovered individuals; RCT: randomized controlled (clinical) trials
[a] parameter source: mixture – mixture of calibration, assumed by authors, and estimated from other data
[b] explicitly mentioned in remarks: if model validation was done; if source code for model is available

References

Arnold ME, Irvine RM, Tearne O, Rae D, Cook AJ, Breed AC: Investigation into sampling strategies in response to potential outbreaks of low pathogenicity notifiable avian influenza initiated in commercial duck holdings in Great Britain. *Epidemiol Infect* 2013, 141:751-762.

Atlas RS, DuPont HL, Overall JE: Computer simulation of alternative sampling strategies to estimate risk of infection from Cryptosporidium. *Comput Biol Med* 1993, 23:283-294.

Auranen K, Rinta-Kokko H, Goldblatt D, Nohynek H, O'Brien KL, Satzke C, Simell B, Tanskanen A, Kayhty H: Design questions for Streptococcus pneumoniae vaccine trials with a colonisation endpoint. *Vaccine* 2014, 32:159-164.

Chen HH, Prevost TC, Duffy SW: Evaluation of screening for nasopharyngeal carcinoma. trial design using Markov chain models. *Brit J Cancer* 1999, 79:1894-1900.

Ciccolini M, Donker T, Grundmann H, Bonten MJ, Woolhouse ME: Efficient surveillance for healthcare-associated infections spreading between hospitals. *P Natl Acad Sci USA* 2014, 111:2271-2276.

Clermont G, Bartels J, Kumar R, Constantine G, Vodovotz Y, Chow C: In silico design of clinical trials. a method coming of age. *Crit Care Med* 2004, 32:2061-2070.

Cori A, Ayles H, Beyers N, Schaap A, Floyd S, Sabapathy K, Eaton JW, Hauck K, Smith P, Griffith S, Moore A, Donnell D, Vermund SH, Fidler S, Hayes R, Fraser C, Hptn PopART Study Team: HPTN 071 (PopART). a cluster-randomized trial of the population impact of an HIV combination prevention intervention including universal testing and treatment: mathematical model. *PloS One* 2014, 9:e84511.

Cuadros DF, Abu-Raddad LJ, Awad SF, Garcia-Ramos G: Use of agent-based simulations to design and interpret HIV clinical trials. *Comput Biol Med* 2014, 50:1-8.

Dimitrov DT, Masse BR, Boily M: Beating the placebo in HIV prevention efficacy trials. the role of the minimal efficacy bound. *J Acq Immun Def Synd* 2013, 62:95-101.

Gonzales JL, Boender GJ, Elbers AR, Stegeman JA, de Koeijer AA: Risk based surveillance for early detection of low pathogenic avian influenza outbreaks in layer chickens. *Preventive veterinary medicine* 2014, 117:251-259.

Graat EA, de Jong MC, Frankena K, Franken P: Modelling the effect of surveillance programmes on spread of bovine herpesvirus 1 between certified cattle herds. *Vet Microbiol* 2001, 79:193-208.

Hallett TB, Garnett GP, Mupamberiyi Z, Gregson S: Measuring effectiveness in community randomized trials of HIV prevention. *Int J Epidemiol* 2008, 37:77-87.

Hayes R, Ayles H, Beyers N, Sabapathy K, Floyd S, Shanaube K, Bock P, Griffith S, Moore A, Watson-Jones D, Fraser C, Vermund SH, Fidler S, Team HS: HPTN 071 (PopART). Rationale and design of a cluster-randomised trial of the population impact of an HIV combination prevention intervention including universal testing and treatment - a study protocol for a cluster randomised trial. *Trials* 2014, 15:57.

Herzog SA, Low N, Berghold A: Sample size considerations using mathematical models. an example with Chlamydia trachomatis infection and its sequelae pelvic inflammatory disease. *BMC Infect Dis* 2015, 15:233.

Hoad KA, van't Hoog AH, Rosen D, Marston B, Nyabiage L, Williams BG, Dye C, Cheng, RCH: Modelling local and global effects on the risk of contracting Tuberculosis using stochastic Markov-chain models. *Math Biosci* 2009, 218:98-104.

Leslie E, Cowled B, Graeme Garner M, Toribio JA, Ward MP: Effective surveillance strategies following a potential classical Swine Fever incursion in a remote wild pig population in North-Western Australia. *Transbound Emerg Dis* 2014, 61:432-442.

Lipsitch M: Measuring and interpreting associations between antibiotic use and penicillin resistance in Streptococcus pneumoniae. *Clin Infect Dis* 2001, 32:1044-1054.

Longini I, Hudgens MG, Halloran ME, Sagatelian K: A Markov model for measuring vaccine efficacy for both susceptibility to infection and reduction in infectiousness for prophylactic HIV vaccines. *Stat Med* 1999, 18:53-68.

Michael E, Malecela-Lazaro MN, Maegga BT, Fischer P, Kazura JW: Mathematical models and lymphatic filariasis control. monitoring and evaluating interventions. *Trends Parasitol* 2006, 22:529-535.

Mizumoto K, Ejima K, Yamamoto T, Nishiura H: On the risk of severe dengue during secondary infection. a systematic review coupled with mathematical modeling. *J Vector Dis* 2014, 51:153-164.

Nishiura H, Yen H, Cowling BJ: Sample size considerations for one-to-one animal transmission studies of the influenza A viruses. *PloS One* 2013, 8:e55358.

Pinsent A, Blake IM, White MT, Riley S: Surveillance of low pathogenic novel H7N9 avian influenza in commercial poultry barns. detection of outbreaks and estimation of virus introduction time. *BMC Infect Dis* 2014, 14:427.

Savill NJ, St Rose SG, Woolhouse ME: Detection of mortality clusters associated with highly pathogenic avian influenza in poultry. a theoretical analysis. *J R Soc Interface* 2008, 5:1409-1419.

Scott P, Herzog SA, Auranen K, Dagan R, Low N, Egger M, Heijne JCM: Timing of bacterial carriage sampling in vaccine trials. a modelling study. *Epidemics* 2014, 9:8-17.

Smieszek T, Salathe M: A low-cost method to assess the epidemiological importance of individuals in controlling infectious disease outbreaks. *BMC Med* 2013, 11.

Tuite AR, Fisman DN: Spectrum bias and loss of statistical power in discordant couple studies of sexually transmitted infections. *STD* 2011, 38:50-56.

van Bunnik BAD, Ciccolini M, Gibbons CL, Edwards G, Fitzgerald R, McAdam PR, Ward MJ, Laurenson IF, Woolhouse ME: Efficient national surveillance for health-care-associated infections. *BMC Public Health* 2015, 15:832.

Vinh DN, Boni MF: Statistical identifiability and sample size calculations for serial seroepidemiology. *Epidemics* 2015, 12:30-39.

Wu HL, Ding AA: Design of viral dynamic studies for efficiently assessing potency of Anti-HIV therapies in AIDS clinical trials. *Biometrical J* 2002, 44:175-196.

Wu HL: Statistical methods for HIV dynamic studies in AIDS clinical trials. *Stat Methods Med Res* 2005, 14:171-192.
